# Low back pain in patients hospitalised with liver cirrhosis- a retrospective study

**DOI:** 10.1186/s12891-023-06424-8

**Published:** 2023-04-19

**Authors:** R. Bednár, D. Líška, D. Gurín, J. Vnenčaková, A. Melichová, T. Koller, Ľ. Skladaný

**Affiliations:** 1 Department of Physiatry, Balneology and Rehabilitation of the Slovak Medical University, F. D. Roosevelt Teaching Hospital, Banská Bystrica, Slovakia; 2 Faculty of Arts, Department of Physical Education and Sports, Matej Bel University, Tajovského 40, Banská Bystrica, 974 01 Slovakia; 3 Faculty of Health Care, Slovak Medical University, Banská Bystrica, Slovakia; 4 2nd Department of Internal Medicine of the Slovak Medical University Faculty of Medicine, HEGITO (Div Hepatology, Gastroenterology and Liver Transplant), F. D. Roosevelt Teaching Hospital, Banská Bystrica, Slovakia; 5 Gastroenterology and Hepatology Subdivision, 5th Department of Internat Medicine, Comenius University, University Hospital Bratislava, Bratislava, Slovakia

**Keywords:** Liver cirrhosis, Lower back pain, Ascites, Stibor, Schober

## Abstract

**Background:**

Lower back pain is a common issue, but little is known about the prevalence of pain in patients with liver cirrhosis during hospitalisation. Therefore, the objective of this study was to determine lower back pain in patients with liver cirrhosis.

**Methods:**

The sample consisted of patients with liver cirrhosis (*n* = 79; men *n* = 55; women *n* = 24; mean age = 55.79 ± 12.52 years). The hospitalised patients were mobile. The presence and intensity of pain were assessed in the lumbar spine during hospitalisation. The presence of pain was assessed using the visual analogue pain scale (0–10). The range of motion of the lower spine was assessed using the Schober and Stibor tests. Frailty was measured by Liver Frailty Index (LFI). The condition of liver disease was evaluated using The Model For the End-Stage Liver Disease (MELD) and Child–Pugh score (CPS) and ascites classification. Student’s t test and Mann–Whitney test were used for analysis of the difference of group. Analysis of variance (ANOVA) with the Tukey post hoc test was used to test differences between categories of liver frailty index. The Kruskal–Wallis test was used to test pain distribution. Statistical significance was determined at the α-0.05 significance level.

**Result:**

The prevalence of pain in patients with liver cirrhosis was 13.92% (*n* = 11), and the mean intensity of pain according to the visual analogue scale was 3.73 (± 1.90). Lower back pain was present in patients with ascites (15.91%; *n* = 7) and without ascites (11.43%; *n* = 4). The prevalence of lower back pain was not statistically significant between patients with and without ascites (*p* = 0,426). The base of Schober’s assessment mean score was 3.74 cm (± 1.81), and based on Stibor’s assessment mean score was 5.84 cm (± 2.23).

**Conclusion:**

Lower back pain in patients with liver cirrhosis is a problem that requires attention. Restricted spinal mobility has been reported in patients with back pain, according to Stibor, compared to patients without pain. There was no difference in the incidence of pain in patients with and without ascites.

## Introduction

Liver cirrhosis is the final stage of chronic liver disease (CLD [1–3]. Slovakia has a high prevalence of liver cirrhosis, which is also the number one cause of death in young adults [4]. In addition to the burden associated with mortality, cirrhosis is associated with repeated hospitalisations for various reasons [5]. The most common causes of CLD and liver cirrhosis in the West are alcohol-associated liver disease (ALD), viral hepatitis B and C (HBV, HCV), and autoimmune liver syndromes, while the most growing is metabolic-associated fatty liver disease (MAFLD), previously known as non-alcoholic fatty liver disease (NAFLD) [6–8]. Morbidity and mortality of cirrhosis are driven by decompensating events (infections, bleeding associated with portal hypertension, malnutrition, sarcopenia, frailty, kidney injury, ascites, and liver encephalopathy). Depending on the onset, these complications lead to acute or chronic decompensation [9]. One of the most frequent specific complications of cirrhosis is ascites, an accumulation of fluid in the peritoneal cavity (Greek askos = water-filled leather bag) [10–12]. Ascites are classified according to their volume into three grades; those of grade three can interfere with activities of daily living and can limit bodily movements [3, 13].

Furthermore, the quality of life of patients with cirrhosis can be reduced by pain, which is often chronic and of musculoskeletal origin [14]. Because of cirrhosis-associated sarcopenia [15–19] and its impact on spine symmetry, a higher burden of low back pain (LBP) could be expected. With a prevalence of 23%, LBP has been one of the most common problems in primary care [15, 16]. The etiopathogenesis of LBP is multifactorial [17–22]. However, despite patient-reported results, the burden of LBP is composed of loss of work productivity, loss of personal income, direct and indirect healthcare expenditures on medical services, surgery, rehabilitation, and pain treatment [23]. Furthermore, there is evidence to suggest that in the West, the burden of chronic LBP has increased [24].

Taking into account the prevalence of both cirrhosis and LBP, it is surprising that little evidence is available on LBP in patients with liver cirrhosis [25]. Therefore, the objective of our study was to analyse a sub-cohort of patients considered for liver transplantation. Our main objective was to determine the frequency of LBP and to examine the associations of LBP with disease-related variables.

## Materials and methods

In our study, a retrospective analysis of the prehabilitation subset of our cirrhosis registry RH7 was conducted [26]. In HEGITO, Division of Hepatology, Gastroenterology, and Liver Transplantation, we have maintained a registry of adult patients admitted to the hospital with liver cirrhosis since 2014 (NCT04767945). For the purpose of this analysis, patients enrolled in RH7 between January 2018 and December 2020 were included. Patients who provided their informed written consent and they did not have standard contraindication were selected in the study. Patients with grade 2 + hepatic encephalopathy, fever, acute skeletal or muscle injury, or flare-up of arthritis involving weight-bearing joints were not included in the study. Cirrhotic patients with interference neurological syndromes, such as central paresis, significant residua after stroke, Parkinson’s disease, sclerosis multiplex, and muscle dystrophy, were not excluded. RH7 was registered at ClinicalTrials.gov under ID NCT04767945.

### Presence of pain

LBP was assessed using a 10-point visual analogue scale (VAS) with 0 meaning no pain and 10 meaning unbearable pain [27]. As a part of the protocol, variables were determined at admission. The presence of LBP was recorded if the pain appeared currently or within the last week.

Spine dynamics were examined using the Schober and Stibor tests. All examinations were performed by an investigator, a senior consultant MD, with experience in physiotherapy of patients with liver cirrhosis.

### Liver Frailty Index (LFI)

The Liver Frailty Index is a core diagnostic modality of the guideline-recommended toolkit for physical frailty [28]. It is characterised by its brevity, validity, and use of only objective parameter – a hand-grip strength (HGS), chair-stand test, and balance test in three postures (side-by-side, semi-tandem, tandem). The original cut-off values were used as determined in the liver transplant setting: LFI < 3.2 = Robust; LFI between 3.2 and 4.4 = Pre-frail; and LFI 4.5 = Frail [29]. Patients were classified according to the liver frailty index as robust, pre-frail, or frail, according to a freely available web calculator (https://liverfrailtyindex.ucsf.edu/) [29]. The baseline characteristics are shown in Table 1.

**Table 1:** Descriptive characteristics of both groups

**Variable**	**N (%)**
**N**	79 (100)
**Male**	55 (69.7)
**Female**	24 (30.3)
	**Mean** ± **SD**
**Age [year]**	55.79 ± 12.52
**Height [cm]**	173.04 ± 7.78
**Weight [kg]**	80.58 ± 18.21
**BMI [kg/m**^**2**^**]**	26.78 ± 5.65
**Aetiology**	**N (%)**
**ALD**	42 (53.16%)
**NASH**	8 (10.13%)
**HBV, HCV**	8 (10.13%)
**PBC, PSC**	8 (10.13%)
**other**	13 (16.45%)
	**Mean** ± **SD**
**MELD**	17.38 ± 6.34
**CHP**	8.03 ± 2.27
**LFI**	4.18 ± 0.96
**Ascites [grade]**	1.09 ± 1.17
**CRP**	19.17 ± 24.85
**LBP**	0.53 ± 1.47
**Stibor [cm]**	5.84 ± 2.23
**Schober [cm]**	3.74 ± 1.81

*BMI* Body mass index, *ALD* Alcohol liver disease, *NASH* Nonalcoholic steatohepatitis, *HBV* Hepatitis B, *HCV* Hepatitis C, *PBC* Primary biliary cholangitis, *PSC* Primary sclerosing cholangitis, *MELD* The Model For the End-Stage Liver, *CHP* Child–Pugh score, *LFI* Liver frailty index, *CRP* C-Reactive Protein, *LBP* Low back pain

### The Model for the End-Stage Liver Disease (MELD) score

Originally, the MELD score was developed to predict prognosis in a particular clinical setting, but, due to its applicability, objectivity, and prediction power, it has gained acceptance as a predictor of survival in cirrhosis in general [30, 31]. The score has been based only on objective parameters and is predictive of survival at a higher value (from an interval of 6–40), meaning a worse prognosis. For example, patients with MELD over 15 should be considered for liver transplantation, since MELD over 21 represents a severe variant of alcoholic hepatitis.

### Child–Pugh score (CPS) and ascites classification

This score, developed some four decades prior to MELD, aimed at the same goal – predicting survival in cirrhosis [32]. In contrast to a purely laboratory-based MELD, incorporated into CPS are also clinically assessed parameters, namely ascites and encephalopathy. The final score is expressed by both a class number and a score/number: CPS class A/5 points = compensated, CPS B/8 = mild decompensation, and CPS C/15 = severe cirrhosis decompensation. Ascites, the accumulation of a fluid in the abdomen, was graded nil if it was not present by ultrasound, grade 1 if detected by ultrasound only (not apparent on physical examination), grade 2 if detectable on physical examination and confirmed by ultrasound, and grade 3 or tense if the skin of the distended abdomen was tight.

### Schober test

**Fig. 1:**
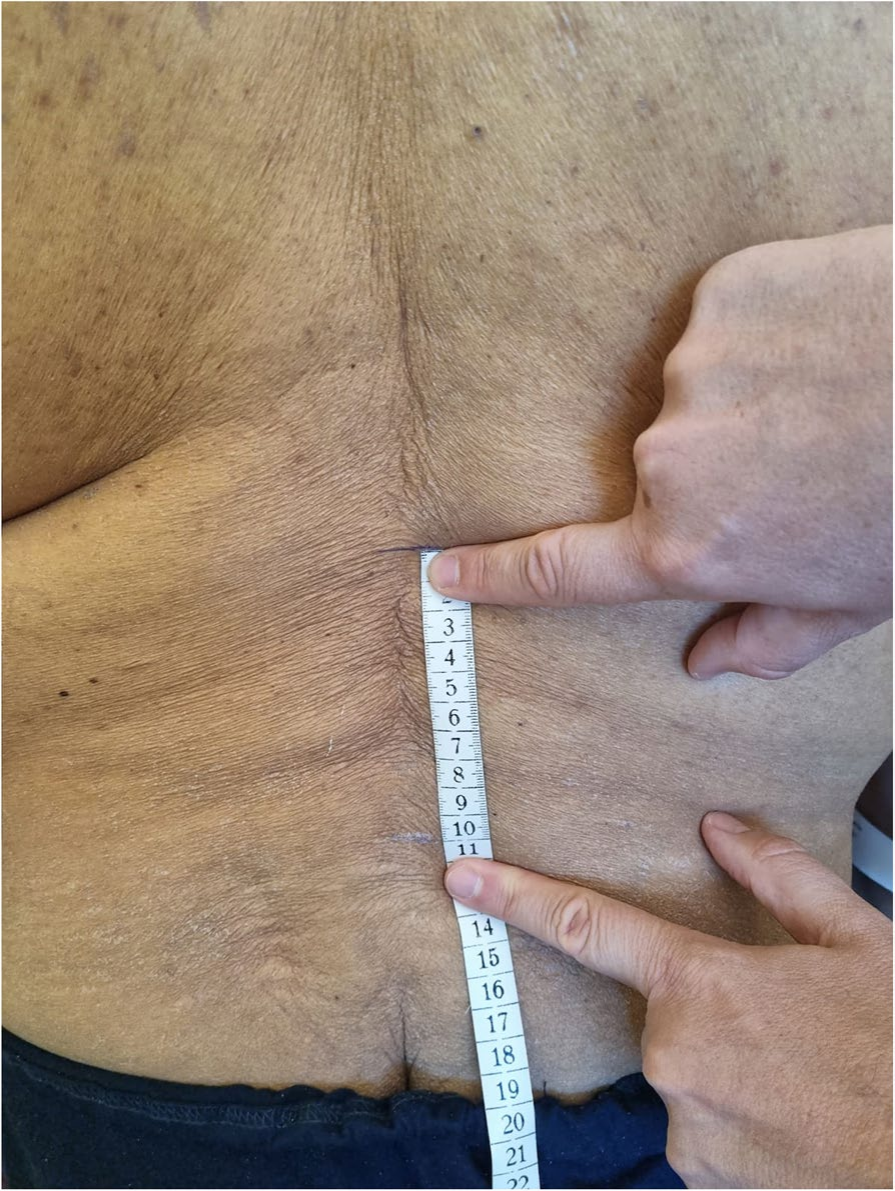
Schober test. Basic assessment position

**Fig. 2:**
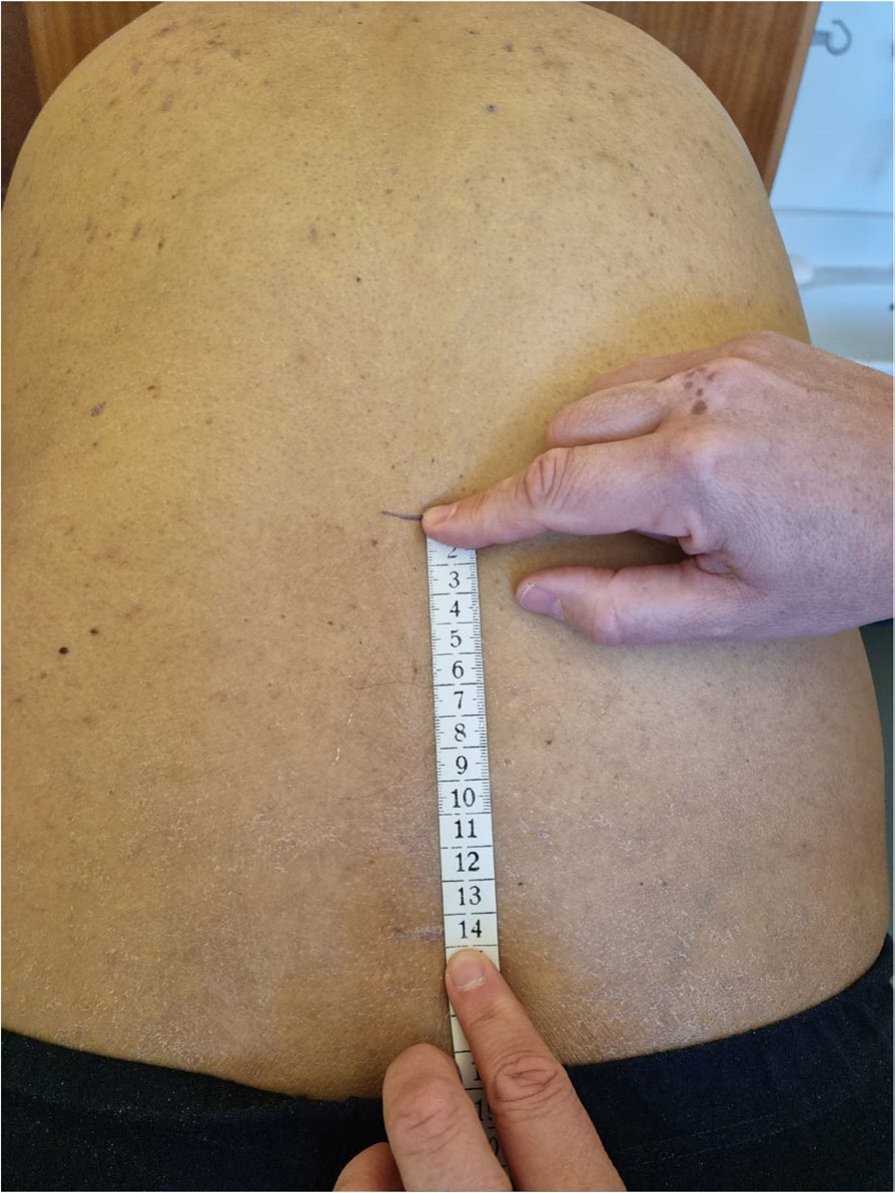
Schober test. Flexion position

This classical test is designed to determine a range of flexion of the lumbar spine. The result is obtained as a detraction of two measurements, both starting at a level of the L5 spinous process. The first step is to draw a horizontal line that crosses the spine 10 cm proximal to the index (Fig. 1). The second is to measure its distancing during maximal forward flexion (Fig. 2). The accuracy of a measurement is to one centimetre.

### Stibor test

The so-called Stibor’s distance examines the unfolding of both the lumbar and thoracic spine’s unfolding during maximal forward flexion. To this end, the second line is set at the level of the C7 spinal process, while the index point is the same as in Schober’s test. The measurements are identical to Schober’s test.

### The sample

Our study included 79 patients (male n-55 (69.7%), female n-24 (30.3%). Hospitalized patients were mobile. Median age of for both groups was 55.79 (± 12.52) years. The median body mass index (BMI) was 26.78 (± 5.65) kg/m^2^ overall, 27.51 (± 5.42) kg/m^2^ in males, and 25.11 (± 6.05) kg/m^2^ in females.

### Statistical analysis

Statistical analysis All baseline characteristics were recorded in MS Excel 2016®. Descriptive statistics and confirmation of differences between the values obtained were performed in XLSTAT®. The normality of the data was confirmed by the Shapiro–Wilk. To verify the statistical significance of the difference, two sample Student’s t-test and Mann–Whitney test were used. Mann–Whitney U test was utilized in non-parametric, abnormal distribution independent groups. Student’s t test was used while using normal distribution was present. Analysis of variance (ANOVA) with the Tukey post hoc test was used to test differences between categories of liver frailty index. The Kruskal–Wallis test was used to test pain distribution. Statistical significance was determined at the α-0.05 significance level.

## Results

In our study, 79 enrolled patients had a mean age of 55.79 (± 12.52), and there were 55 males (69.7%) and 24 females (30.3%). Of the patients, 42 had cirrhosis caused by ALD (53.2%), and the baseline MELD was 17.38 points (± 6.34) Table 1). The mean LFI was 4.18 (± 0.96). Frailty was present in 22 (27.84%) patients (Table 3). At the time of examination, 7 out of 79 patients (8.86%) used painkillers to other causes.

Eleven of 79 patients (13.9%) reported LBP according to the study criteria (Table 2). Their median age was 55.40 (± 13.95) years. The age of patients with and without LBP was not statistically significant (*p* = 0.424). The Pain Intensity Score by VAS was mild 3.73 (± 1.90) points (men 3.57 ± 1.72; women 4.00 ± 2.45; *p* > 0.05). Ascites were present in 44 patients (55.70%). LBP was present in 7 patients (15.91%) with ascites and in four patients (11.43%) without ascites (*p* = 0.426). The pain intensity scores in patients with and without ascites were 3.43 (± 2.15) and 4.25 (± 1.50), respectively (*p* = 0.1638).

**Table 2:** Comparison of patients with and without LBP

**Variable**	**With LBP (*n* = 11)**	**Without LBP (*n* = 68)**	*p*-value*
	Mean ± SD	Mean ± SD	
**Age [year]**	55.40 ± 13.95	55.79 ± 12.82	0.424
**BMI [kg/m**^**2**^**]**	27.05 ± 5.07	26.76 ± 5.81	0.435
**Height [cm]**	173.45 ± 7.09	172.97 ± 7.99	0.318
**Weight [kg]**	81.45 ± 16.63	80.44 ± 18.70	0.492
**MELD**	17.00 ± 7.10	17.44 ± 6.31	0.417
**CHP**	7.80 ± 2.44	7.94 ± 2.24	0.369
**LFI**	4.24 ± 0.53	4.09 ± 0.94	0.180
**Ascites [grade]**	0.90 ± 0.99	1.03 ± 1.16	0.426
**Stibor [cm]**	4.68 ± 2.8	6.08 ± 2.09	0.028*
**Schober [cm]**	3.40 ± 1.07	3.83 ± 1.94	0.118

*SD* Standard deviation, *BMI* Body mass index, *LBP* Low back pain, *MELD* The Model For the End-Stage Liver Disease, *CHP* Child–Pugh score, *LFI* Liver frailty index

The mean Schober’s distance was 3.74 cm (± 1.81) in both groups and did not differ between patients with and without ascites (*p* = 0.1637). The mean Stibor test distance was 5.84 cm (± 2.23) in both groups. A significant difference was found between patients with LBP and those without LBP according to the Stibor test (*p* = 0.028). Spine mobility by Schober and Stibor tests was decreased in frail patients (Tables 3 and 4).

**Table 3:** Baseline characteristics according to the liver frailty index

	**Age**	**BMI**	**Schober**	**Stibor**	**MELD**	**CHP**
**Robust** (*n* = 10)						
**Mean**	44.44	22.92	4.44	7.78	16.67	7.11
**SD**	10.92	4.43	0.58	0.71	4.80	1.76
**Prefrail** (*n* = 47)						
**Mean**	56.96	27.24	4.03	5.94	16.29	7.56
**SD**	11.93	5.48	2.17	2.22	6.91	2.19
**Frail** (*n* = 22)						
**Mean**	58.21	27.40	2.81	4.66	19.84	9.16
**SD**	13.72	6.72	0.77	1.97	5.36	2.22

*SD* Standard deviation, *BMI* Body mass index, *MELD* The Model For the End-Stage Liver Disease, *CHP* Child–Pugh score

**Table 4:** Low back pain and mobility of the spine according to the presence of frailty

(*Sig 0.05)	**LBP**	**Schober**	**Stibor**
**Robust vs prefrail**	0.558	0.789	0.012*
**Robust vs frail**	0.376	0.060	0.000*
**Frail vs prefrail**	0.824	0.040*	0.067

## Discussion

The motivation behind our focus on LBP in patients with liver cirrhosis is that LBP is a considerably prevalent and consequential condition in the general population. It is scarcely inspected in cirrhosis, and most importantly, there are several reasons to suspect that the risk and consequences of cirrhosis increase. First, the prevalence of LBP in a general population is associated with increasing age, and frailty cirrhotic patients are considered some 15 years biologically older than their chronological age; therefore, a question arises: Is this factor translated into an increased impact of LBP in cirrhosis? Second, frailty associated with cirrhosis, sarcopenia, inactivity, motor neuronal impairment, and ascites are possible risk factors that contribute to impaired mobility of the spine and LBP. These and other links between cirrhosis, the lumbar spine, and LBP, together with the scarce literature on the subject, have become the inspiration for the current study.

Eleven patients (13.9%) with LBP were identified. Treatment of pain in patients opens the door to the perception of an underexplored area with the potential to affect the outcome of cirrhosis. Although derived from a cohort size and of a relatively low VAS intensity (3.73 ± 1.90), a long-term LBP represents a serious threat to mobility, muscle mass, and function, as well as the quality of life of our patients. These factors can accumulate and, over time, lead to a bad outcome in terms of dropping out of the waiting list for a liver transplant, a worse outcome of LT, and mortality. 

Identifying a new association of these clinical factors with pain would have the potential to focus more / personalised attention on physiotherapy. Therefore, this could lead to improved quality of life, adherence to physiotherapy advice, and eventually increased survival to liver transplant, as well as overall survival [33].

In contrast to our expectations, no increased prevalence of LBP was found in patients with ascites. We based our assumption on the influence of the static and dynamic aspects of spine physiology. The psoas muscles also supported our hypothesis of an increase in LBP in ascitic cirrhosis because, on the one hand, they are the main supporting apparatus, and, on the other hand, they are the primary site of sarcopenia in cirrhosis [34, 35]. Sarcopenia is often present in patients with liver cirrhosis. Sarcopenia is characterised by an involuntary loss of muscle mass and function [36]. However, the consequences of sarcopenia are much greater than the decline in functional capacity and include a number of adverse health effects [35, 37]. Liver transplant sarcopenia is associated with poorer outcomes, including a reduced survival rate [34].

Patients with more advanced liver disease have an increased prevalence of pain [38], which is associated with sleep and mood disorders, as well as a high risk of disability, but the incidence of LBP was not significant in our cohort. Madan et al. [39] evaluated the incidence of chronic pain in patients with end-stage liver cirrhosis before transplantation. Of the patients, 77% reported pain in some parts of the body within 24 h, 90% reported taking some type of analgesic, and 32% reported acute pain throughout the spine within 24 h. The average pain intensity on the visual analogue scale was 4.25. Compared to our study, the incidence of back pain was significantly higher.

The association of pain with Stibor and Schober tests can be formulated as the more limited the movement of the spine, the more pain patients experience. This is important since the performance of Stibor and Schober is feasible and safe in real-life clinical practice, and when testing positive (limited range), patients can be motivated by more advice to increase their flexibility.

Physical performance has also been found to be associated with altered spine dynamics by other authors [40]. Furthermore, by the same token, our results offer a new dimension of physical frailty in people with cirrhosis as an indirect marker of the health status of the spine. This can be used to the advantage of our patients by submitting them to physiotherapy earlier than before. Currently, LFI-triggered physiotherapy has focused primarily on muscle mass and performance, with spine mobility not specifically addressed. It would be interesting to investigate the impact of including the new pathway *frailty – spine – pain* in the current approach based on the pathway *frailty – muscle – prognosis.* It could be that such modified physical therapy will result in a better quality of life, adherence, and prognosis for patients with cirrhosis (especially ascites) [41]. Based on our results, we cannot exclude the possibility that decreased spine mobility is related to the relatively high age of our patients, which is a well-known risk factor. However, even if confirmed in future studies, the consequences of physical therapy should not differ.

Compared to other diseases, the incidence of LBP was low in patients with liver cirrhosis. Patients with COPD suffer from nonspecific LBP, which varies from 41.2% to 69% [42, 43] in cardiovascular diseases; the prevalence of LBP is approximately 36.6% [43] in metabolic syndrome, and LBP occurs in 25% [44, 45]. A high BMI is a significant risk factor for LBP in patients with liver cirrhosis. Obesity is a common public health problem [46] and a significant risk factor for LBP [47]. Obesity is considered a risk factor for liver disease [48]. Increased body weight also affects posture. Higher lumbar spine hyperlordosis can be observed in overweight and obese patients [49, 50]. Lumbar spine hyperlordosis is a risk factor for back pain [51], and in patients with liver cirrhosis, it can be associated with back pain.

In general, patients with liver cirrhosis are less physically active than patients without liver cirrhosis. Hypoactivity is considered one of the most important factors in the development of LBP. The purpose of the meta-analysis by Alzahrani et al. [52] was to examine the association between total physical activity and LBP in adults. The study found an inverse association between movement activity and LBP. Moderate levels of physical activity were associated with a lower prevalence of LBP. Lower physical activity is common in patients with liver cirrhosis [53]. This low physical activity can be related to a fear of movement and deterioration. This barrier may contribute to a higher prevalence of pain associated with hypoactivity.

Treating LBP in patients with liver cirrhosis can be difficult. In most cases, conservative therapies are used to treat LBP [54]. Rehabilitation therapies are used in most cases [55], but there is a lack of studies on the efficacy of therapies in patients with liver cirrhosis.

Therefore, before fully appreciating the neutral association between ascites and LBP, more data are needed from other studies with more patients. If neutrality was confirmed, this could be explained by the decreased mobility of patients with ascites, where pain is less likely to be elicited.

In our study, 7 patients took analgesics for other causes, which could have affected the incidence and intensity of pain in patients. The use of analgesics in patients with liver cirrhosis is common [56]. According to Rogal et al. [38], 25% of patients with cirrhosis pain use opioids. The use of analgesics and opioids at the time of examination could lead to biased results and reduced pain in patients.

Acute spinal pain was followed during hospitalisation in patients with liver cirrhosis who remained present for one week. However, if we had followed the incidence in the past, the incidence in patients would probably be much higher.

Another limitation was the size of the group, despite the larger group of patients, with a larger examination, it is possible to see a higher or lower incidence of back pain in patients with liver cirrhosis.

## Conclusion

LBP in patients with liver cirrhosis is a problem that requires attention. However, our study did not report a higher incidence of LBP. Restricted spinal mobility has been reported in patients with back pain in Stibor test compared to patients without pain. There was no difference in the incidence of pain in patients with ascites and without ascites.

## Data Availability

The datasets used and/or analysed during the current study available from the corresponding author on reasonable request.
